# The role of Shenqi Fuzheng injection as adjuvant therapy for breast cancer: an overview of systematic reviews and meta-analyses

**DOI:** 10.1186/s12906-023-04274-4

**Published:** 2024-01-11

**Authors:** Jing Xu, Xiao Li, Liyuan Lv, Qing Dong, Xiaofeng Du, Guangda Li, Li Hou

**Affiliations:** 1 Department of Hematology and oncology, Dongzhimen Hospital Affiliated with, Beijing University of Chinese Medicine, Beijing, China; 2 Beijing University of Chinese Medicine, Beijing, China

**Keywords:** Traditional Chinese medicine, Shenqi Fuzheng injection, Breast cancer, Meta-analyses, Systematic review

## Abstract

**Background:**

Breast cancer (BC) is the most frequent malignancy in the world. Chemotherapy (CT) is a common treatment for BC but is accompanied by toxicity and side effects. Shenqi Fuzheng Injection (SFI) is an adjuvant therapy with promising results in improving efficacy and reducing toxicity in clinical studies. This overview of systematic reviews and meta-analysis (SRs/MAs) aimed to summarize the benefits and evaluate the quality of evidence supporting SFI adjuvant as CT for BC.

**Methods:**

A systematic search for SRs/MAs of randomized controlled trials (RCTs) on SFI treatment for BC was performed by searching PubMed, Web of Science, EMbase, Cochrane Library, CNKI, Wanfang, VIP, and SinoMed databases from inception to October 1, 2022. The quality of SRs/MAs was evaluated using AMSTAR-2, PRISMA 2020, ROBIS, and GRADE by two reviewers. The corrected covered area (CCA) was used to quantify the degree of duplication of the original SRs/MAs. Finally, quantitative analysis of RCTs was conducted using RevMan 5.4 software. This study was registered with PROSPERO, CRD42022377290.

**Results:**

Six SRs/MAs including 61 RCTs with 5593 patients were included in this study. Studies were published between 2015 and 2019, the original RCTs ranged from 7–49, with sample sizes ranging from 336–1989. The quantitative meta-analysis found that adjuvant CT of SFI improved the clinical response rate (RR=1.37, 95% CI=1.28, 1.46; *P*<0.00001) and the KPS score (RR=1.66, 95% CI 1.54, 1.79, *P*<0.00001) of patients with BC. In terms of immune function, CD3+ (SMD=1.51, 95% CI 0.91, 2.10; *P*<0.00001), CD4+ (SMD=1.87, 95% CI 1.18, 2.56; *P*<0.00001), CD4+/CD8+ (SMD=0.86, 95% CI 0.48, 1.23; *P*<0.00001), and NK cell levels (SMD=0.94, 95% CI 0.63, 1.24; *P*<0.00001) in the adjuvant CT group SFI were better than those with CT alone. Adverse reactions following SFI adjuvant CT showed reduced incidence of leukopenia (RR=0.53, 95% CI 0.46, 0.62; *P*<0.00001) and gastrointestinal reactions (RR=0.48, 95% CI 0.39, 0.58; *P*<0.00001). However, the GRADE results showed ‘very low’ to ‘moderate’ evidence for the 42 outcomes, without high-quality evidence supporting them, limited mainly by deficiencies in the design of RCTs (42/42, 100.00%), inconsistency (19/42, 45.24%), publication bias (41/42, 97.62%), and inaccuracy (3/42, 7.14%). The unsatisfactory results of AMSTAR-2, PRISMA 2020, and ROBIS were limited to lack of registration of study protocols, explanation of inclusion basis of RCTs, description of funding sources for the included studies, incomplete search strategy and screening process, addressing heterogeneity and sensitivity, and reporting potential conflicts of interest.

**Conclusion:**

Adjuvant CT with SFI for BC had better benefits and a lower risk of adverse events. The methodology and quality of the evidence are generally low, highlighting a need of greater attention during study implementation. More objective and high-quality studies are needed to verify the efficacy of adjuvant CT with SFI in clinical decision-making for BC.

**Supplementary Information:**

The online version contains supplementary material available at 10.1186/s12906-023-04274-4.

## Background

Breast cancer (BC) is the most common malignancy in women. According to global cancer statistics in 2020, the number of new cases of BC is 2.3 million, representing 11.7% of the new cases of malignant tumors in the world, surpassing lung cancer (11.4%) for the first time and ranking first in the world [1]. Cancer treatment methods are constantly updated and explored, but chemotherapy (CT) is one of the most commonly used treatment methods for advanced BC [2, 3]. CT can easily induce gastrointestinal reactions, bone marrow suppression, liver and kidney damage, and other toxic side effects, which are difficult for patients to tolerate, resulting in the interruption of CT or the reduction of CT dose, thus limiting its application. The identification of safe and effective adjuvant drugs is one of the problems that needs to be solved clinically [4].

The main ingredients of the Shengqi Fuzheng injection (SFI) are Codonopsis pilosula and Astragalus mongholicus. These ingredients have been reported to have antitumor effects (Table 1). Studies have shown that SFI can inhibit tumor progression, reduce surgical complications, improve chemoradiotherapy sensitivity, and improve body immunity [5]. Systematic reviews and meta-analysis (SRs/MAs) have confirmed that adjuvant CT with SFI can improve the clinical efficacy in patients with BC, improve quality of life, regulate body immune function, and reduce adverse reactions [6–8]. SRs/MAs are considered the highest level of evidence in the field of evidence-based medicine, but the value of their evidence depends largely on the quality of the included studies, and SRs/MAs with serious deficiencies in methodological quality can mislead decision makers [9, 10]. Although several SRs/MAs on adjuvant CT therapy with SFI for BC have been published, methodological quality and evidence strength have not been evaluated. Therefore, the objective of this study was to objectively and comprehensively evaluate the systematic review of adjuvant treatment of BC with SFI to determine the efficacy and safety of SFI in the treatment of BC.

**Table 1:** Details of the ingredients of SFI

**Name**	**Chinese name**	**Family**	**Medicinal parts**	**Anti-tumor pharmacological effect**
Codonopsis pilosula	Dangshen	*Campanulaceae*	root	①Inhibit the proliferation, invasion, migration and adhesion of tumor cells [11]; ②2.Promote tumor cell apoptosis [12]; ③Regulate the body's immune function [13–15]; ④Play a synergistic role with chemotherapy drugs [16].
Astragalus mongholicus	Huangqi	*Leguminosae*	root	①Inhibit the proliferation and differentiation of tumor cells [17]; ②Inhibit the invasion and migration of tumor cells [18, 19]; ③Promote tumor cell apoptosis [20]; ④Regulate the body's immune function [21]; ⑤Enhance the sensitivity of chemotherapy drugs [22].

### Materials and methods

#### Protocol and registration

PROSPERO is an international prospective register website of systematic reviews, which accepts registrations for systematic reviews, rapid reviews, and umbrella reviews. The protocol for this review had been registered with PROSPERO prior to beginning the review, and the registration number was CRD42022377290.

### Data sources and literature search

**Fig. 1:**
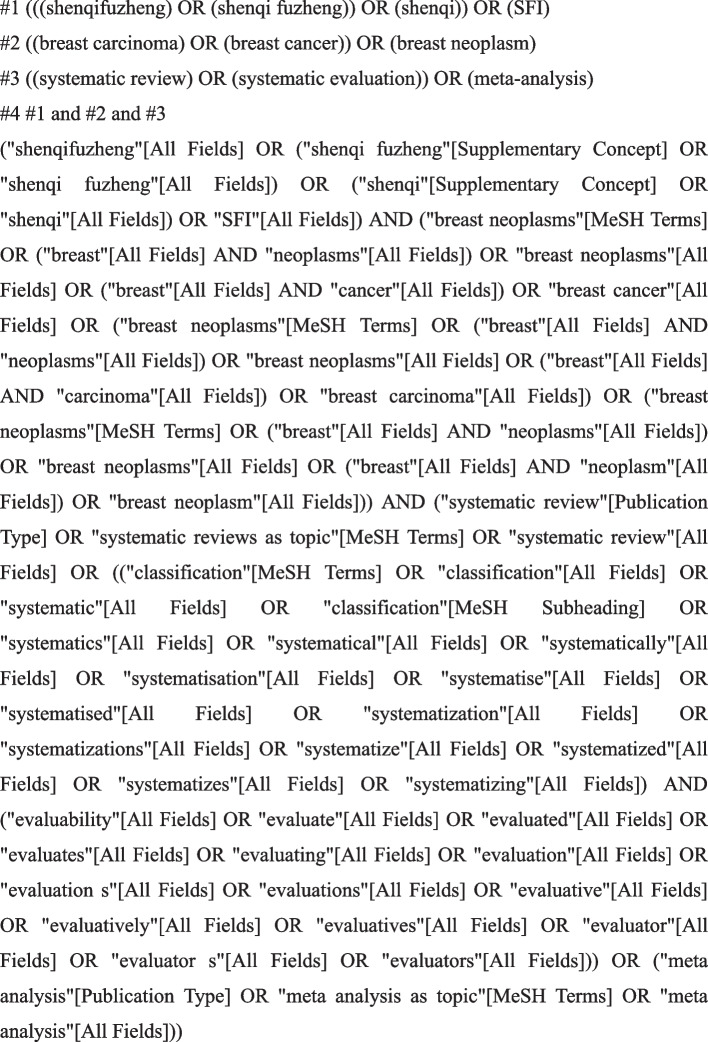
Pubmed retrieval process

Two reviewers (Jing Xu and Xiaofeng Du) searched the following databases from their inception to October 1, 2022. PubMed, Web of Science, EMbase, Cochrane Library, China National Knowledge Infrastructure Database (CNKI), WanFang database, China Science and Technology Journal Database (VIP), and SinoMed. Additionally, all references included in the SRs/MAs were retrieved a second time. The retrieval strategy used a combination of subject words and free words. The PubMed search strategy is presented in detail in Fig. 1. The search strategies of other databases are shown in Supplementary Material 1.

### Inclusion and Exclusion Criteria

The reliability criteria followed the PICOS principles, as follows: (1) participants: patients with BC confirmed by histopathology or cytology, regardless of sex, age, race, and course of the disease; (2) interventions: SFI adjuvant CT, without limiting the CT scheme, frequency, and dose; (3) Comparator(s)/control: CT alone, without limiting the CT scheme, frequency, and dose; (4) outcomes: clinical response rate, KPS score, immune function (CD3+, CD4+, CD8+, CD4+/CD8+, NK cells), adverse reactions (incidence of leukopenia and gastrointestinal reactions); and (5) type of study: as randomized controlled trials (RCT) are the gold standard for evaluating clinical evidence, SRs/MAs based on RCT were selected for the overview, unrestricted language.

We excluded network meta-analysis, studies that included TCM decoction and other TCM therapies in the intervention, studies where full text data was not available, and studies with data errors.

### Literature screening and data extraction

The overview used EndNote X9 to perform a secondary search to remove duplicates and read the remaining literature in depth. SRs/MAs were independently screened by two reviewers (Jing Xu and Liyuan Lv) according to the inclusion criteria, data were extracted and cross-reviewed. Any dispute was resolved by the third reviewer (Hou Li). The authors were contacted when necessary to obtain the complete original data. Two reviewers (Jing Xu and Liyuan Lv) independently extracted data including first author, publication year, language, country, number of the included RCTs, sample size, intervention measures, bias risk assessment tools, outcome indicators, funding, and conflict of interest statements.

### Calculation of repetition rate

Overview of the included SRs/MAs can exaggerate the efficacy of the studies due to excessive overlap of the original literature, leading to similar conclusions about SRs/MAs. Therefore, an overview establishes the overlap matrix of SRs/MAs and its included original literature and calculates the Corrected Covered Area (CCA) to evaluate the degree of duplication of SRs/MAs original literature [23]. The calculation formula is CCA=(Nr)/(rc-r), where N is all original SRs/MAs studies (including duplications), r is all original studies after eliminating duplicates, and c is the number of SRs/MAs included in the overview. If the CCA is 100%, it indicates that every review included in the overview contains the same RCTs, while if the CCA is 0%, it indicates that every review included in the overview contains completely different RCTs [23]. A CCA of ‘0% to 5%’ indicates slight overlap, ‘6% to 10%’ indicates moderate overlap, ‘11% to 15%’ indicates high overlap, and ‘≥15%’ indicates extremely high overlap.

### Evaluation of methodological quality

AMSTAR-2, an internationally recognized systematic methodological quality assessment tool, was used to evaluate the methodological quality of the included SRs/MAs [24]. AMSTAR-2 includes a total of 16 items, items 2, 4, 7, 9, 11, 13, and 15 are critical items, others are noncritical items. Two reviewers (Jing Xu and Xiao Li) independently performed the evaluations. Any dispute was discussed or decided by the third reviewer (Li Hou). When the corresponding content matched an item, it was marked as ‘yes,’ when it did not match completely, it was marked as ‘partial yes,’ and when it did not match, it was marked as ‘no.’ When there was no or only one non-critical item not satisfied, it was rated as ‘high’ quality. If more than one non-critical item was not satisfied, it was rated as ‘moderate’ quality. If only one critical item was not satisfied, it was rated as low-quality. When more than one critical item did not meet the criteria, it was rated as ‘critically low’ quality.

### Evaluation of reporting quality

The statement Preferred Reporting Items for Systematic Review and Meta-analyses (PRISMA) 2020, an updated guideline for reporting systematic reviews, contains 27 items (42 sub items), including seven parts such as title, abstract, preface, method, result, discussion, and others. On completion of SRs/MAs, each item is evaluated as ‘yes,’ ‘no,’ or ‘partially yes’ [25]. The evaluation of the quality of the overview reporting was independently evaluated by two reviewers (Jing Xu and Xiao Li), and any disagreement was discussed or decided by the third reviewer (Li Hou).

### Assessment of risk of bias (ROBIS)

The Risk of Bias in Systematic Reviews (ROBIS) is a tool for assessing the risk of bias of SRs/MAs. The tool is divided into three phases and is mainly used to mark different biases in phases 2 and 3. Phase 2 includes four domains: research identification and selection, data collection and study evaluation; extraction, quality evaluation, synthesis and quality evaluation; and synthesis and results presentation). Instead, phase 3 assesses the overall risk of bias. The degree of SRs/MAS bias risk judgment is expressed as ‘low,’ ‘high,’ and ‘uncertain’ [26]. Two reviewers (Jing Xu and Xiao Li) independently assessed the bias risk of SRs/MAs through ROBIS and cross-checked after completion, and any disagreement was discussed or decided by the third reviewer (Li Hou).

### Evaluation of the quality of evidence

The Grades of Recommendation, Evaluation, Development, and Evaluation (GRADE) are applied for evidence quality assessment of included outcomes using five downgrade factors: study design, imprecision, inconsistencies, indirectivity, and risk of bias. After the assessment, the quality of the evidence was classified into four levels: ‘high,’ ‘moderate,’ ‘low,’ and ‘very low’ [27]. Evaluators are trained to reach consensus before performing an assessment. The two reviewers (Jing Xu and Xiao Li) were trained prior to formal evaluation, and any disagreements were discussed or decided by the third researcher (Li Hou).

### Statistical analysis

To better clarify the efficacy of adjuvant CT of SFI for BC, RevMan 5.4 was used for the quantitative analysis of the included SRs/MAs. Results are reported using the standardized mean difference (SMD), odds ratio (OR) or relative risk (RR), 95% confidence interval (CI), random effects model, fixed effects model, and heterogeneity (I^2^). If *P*>0.1 and I^2^≤ 50%, the fixed effect model is adopted. Instead, if *P*<0.1 and I^2^≥50%, the random effects model was used [28]. If heterogeneity was significant, subgroup or sensitivity analysis was used to reduce heterogeneity, and the funnel plot was used to determine whether publication bias existed in the included studies.

## Results

### Literature screening and descriptive characteristics

**Fig. 2:**
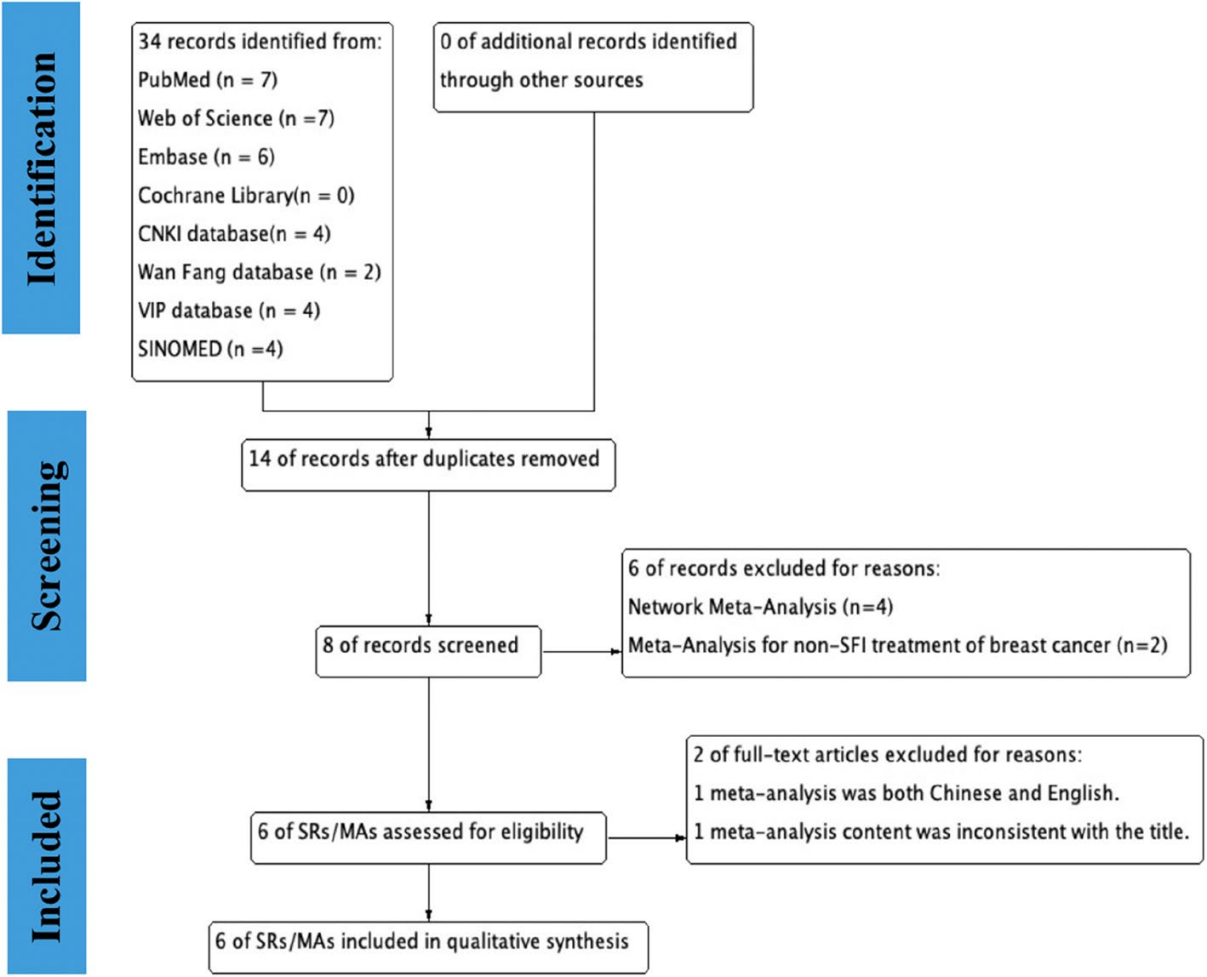
Flow chart of the literature selection process

The process of SRs/MAs retrieval, screening, and selection in the overview was shown in a PRISMA flow chart (Fig. 2). A preliminary search obtained 34 studies, of which 14 were obtained after deleting duplicates, and of these six were excluded due to network meta-analysis and meta-analysis not including SFI for BC. One meta-analysis was repeated in both Chinese and English, and one meta-analysis was excluded due to inconsistent content and title; finally, six SRs/MAs were included in the overall analysis [6–8, 29–31].

Six SRs/MAs including 61 RCTs with 5593 patients were eligible for this study after removing duplicates. The overview provided a table of the main characteristics of six SRs/MAs, including five in English and one in Chinese. The time span included in the overview was 2015–2019 (Table 2). Six SRs/MAs were included in the original RCT range from 7 [29] to 49 [7], and the total sample size was between 542 [29] and 4385 [7]. All original RCTs included in SRs/MAs were developed and implemented in China.

**Table 2:** Characteristics of included SRs/MAs

**Study ID**	**Country**	**Language**	**Study type**	**trials (cases)**	**Intervention(T/C)**		**Quality assessmnet**	**Main results**	**Funding**	**Interest statement**
Yang AL 2018 [29]	China	Chinese	RCT	7(542)	SFI+CT	CT	Cochrane risk of bias tool	①③	N	N
Wu JH 2019 [8]	China	English	RCT	20 (2095)	SFI+CT	CT	Cochrane risk of bias tool	①②③④	N	N
Liu SY 2018 [6]	China	English	RCT	31 (2543)	SFI+CT	CT	Cochrane risk of bias tool	①②③④	Y	Y
Lv YH 2015 [30]	China	English	RCT	18 (1247)	SFI+CT	CT	Cochrane risk of bias tool and JADAD	①②③④	Y	Y
Li Y 2016 [31]	China	English	RCT	20 (1609)	SFI+CT	CT	Cochrane risk of bias tool	①②③④	N	Y
Zhang HB 2019 [7]	China	English	RCT	49 (4385)	SFI+CT	CT	Cochrane risk of bias tool	①②③④	N	Y

*N* NO, *Y* Yes.

*T* Test group, *C* Control group, ①Clinical response rate; ②KPS; ③Immune function (CD3+、CD4+、CD8+、CD4+/CD8+、NK cell); ④ Adverse reactions (leukopenia and gastrointestinal reactions).

### Original literature repetition rate

**Fig. 3:**
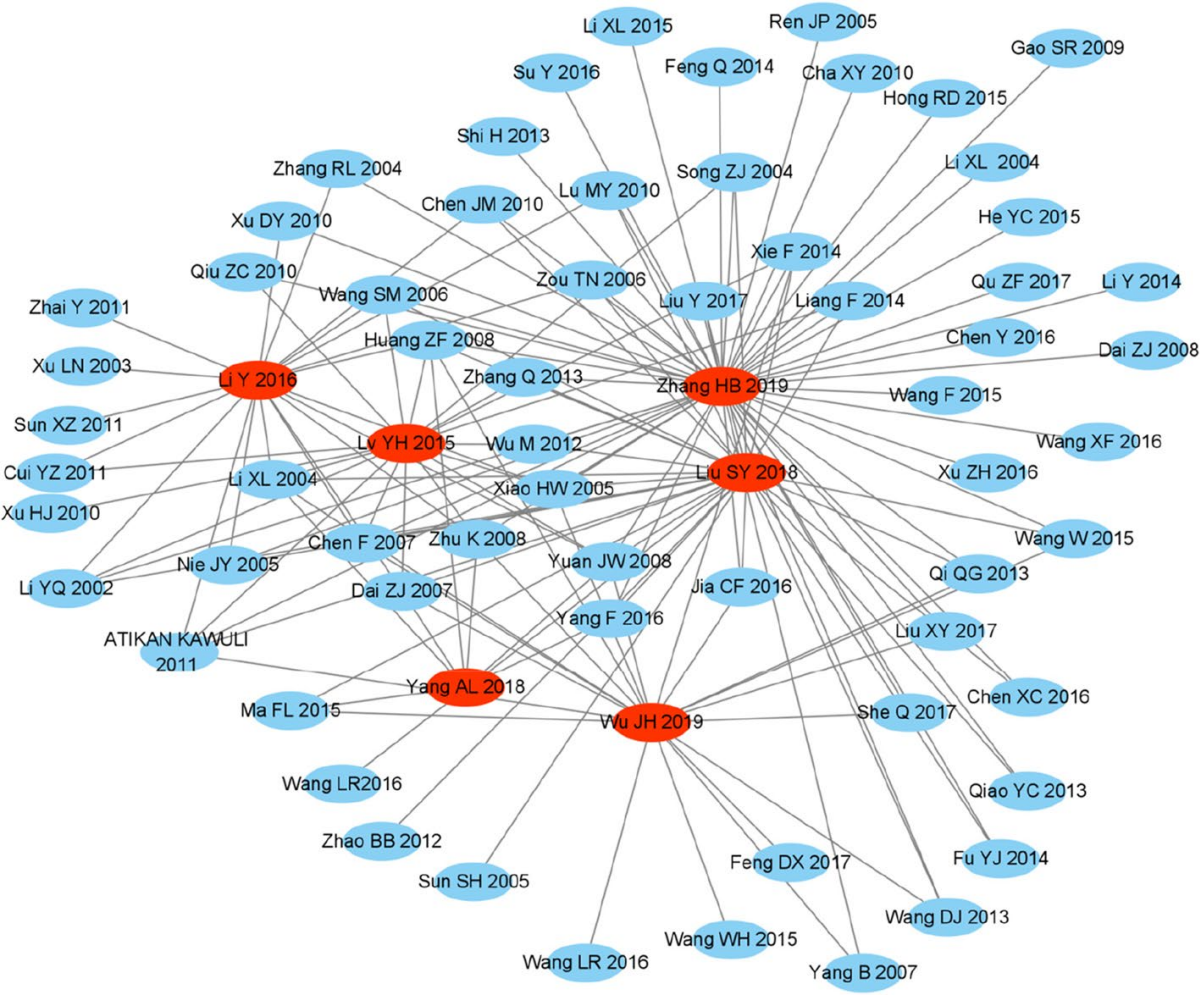
"Cloud" relationship between SRs/MAs and original RCTs

The overview included a total of six SRs/MAs, and the number of all original studies included was 145, 61 after the removal of duplicate literature. According to the formula, N was 145, r was 61, and c was 6, CCA=(145–61)/(61×6–61)*100%=27%, which indicated a large overlap. This reflects the unnecessary duplication of SRs/MAs in adjuvant CT treatment with SFI of BC. Future SRs/MAs should only be conducted when the research objectives are significantly different. Prospective registration of SRs/MAs may help avoid unnecessary duplications [32]. Figure 3 showed the ‘cloud’ relationship between the included SRs/MAs and the original RCTs. The more original the research lines, the more frequently they were included.

### Methodological quality of the included SRs/MAs

Table 3 summarizes the results of the methodological quality assessment of six SRs/MAs in the AMSTAR-2 overview. Because all SRs/MAs presented one or more critical weaknesses, the quality of the methodology used to identify them was low or very low. Three SRs/MAs were of very low quality, and three SRs/MAs were of low quality. Systematic methodological quality reviews varied widely and most of the included studies showed some limitations. For example, none of the studies preregistered the study protocol prior to the systematic review, none explained the basis for including only RCTS in the systematic review, and none reported the source of funding for each included study. Two studies [8, 29] did not use duplicate data extraction by double reviewers and did not report all potential sources of conflict of interest. A study [29] did not provide a list of excluded references. Two studies [29, 30] did not assess the impact of the bias risk of each included study on the results of the SRs/MAs, or did not consider the bias risk of the included study, and did not reasonably explain heterogeneity of the results. Two studies [29, 31] did not adequately investigate publication bias.

**Table 3:** Methodological quality assessment of SRs/MAs by AMSTAR-2

**Reference**	**Yang AL 2018 **[29]	**Wu JH 2019 **[8]	**Liu SY 2018 **[6]	**Lv YH 2015 **[30]	**Li Y 2016 **[31]	**Zhang HB 2019 **[7]	**Number of y (%)**
Question							
Q1	y	y	y	y	y	y	6(100%)
Q2	n	n	n	n	n	n	0(0)
Q3	n	n	n	n	n	n	0(0)
Q4	py	py	y	py	y	y	3(50%)
Q5	y	y	y	y	y	y	6(100%)
Q6	n	n	y	y	y	y	4(66.67%)
Q7	n	y	y	y	y	y	5(83.3%)
Q8	py	y	y	y	y	y	5(83.3%)
Q9	y	y	y	y	y	y	6(100%)
Q10	n	n	n	n	n	n	0(0)
Q11	y	y	y	y	y	y	6(100%)
Q12	n	y	y	n	y	y	4(66.67%)
Q13	n	y	y	n	y	y	4(66.67%)
Q14	n	y	y	n	y	y	4(66.67%)
Q15	n	y	y	y	n	y	4(66.67%)
Q16	n	n	y	y	y	y	4(66.67%)
Overall quality	CL	L	L	CL	CL	L	

Q, question; Y, yes; N, no; PY, partial yes; L, low; CL, critically low.

Q1: Did the research questions and inclusion criteria for the review include the components of PICO?

Q2: Did the report of the review contain an explicit statement that the review methods were established prior to the conduct of the review and did the report justify any significant deviations from the protocol?

Q3: Did the review authors explain their selection of the study designs for inclusion in the review?

Q4: Did the review authors use a comprehensive literature search strategy?

Q5: Did the review authors perform study selection in duplicate?

Q6: Did the review authors perform data extraction in duplicate?

Q7: Did the review authors provide a list of excluded studies and justify the exclusions? Q8: Did the review authors describe the included studies in adequate detail?

Q9: Did the review authors use a satisfactory technique for assessing the risk of bias (RoB) in individual studies that were included in the review?

Q10: Did the review authors report on the sources of funding for the studies included in the review?

Q11: If meta-analysis was performed did the review authors use appropriate methods for statistical combination of results?

Q12: If meta-analysis was performed, did the review authors assess the potential impact of RoB in individual studies on the results of the meta-analysis or other evidence synthesis?

Q13: Did the review authors account for RoB in individual studies when interpreting/discussing the results of the review?

Q14: Did the review authors provide a satisfactory explanation for, and discussion of, any heterogeneity observed in the results of the review?

Q15: If they performed Quantitative synthesis did the review authors carry out an adequate investigation of publication bias (small study bias) and discuss its likely impact on the results of the review?

Q16: Did the review authors report any potential sources of conflict of interest, including any funding they received for conducting the review?

### Reporting quality of included SRs/MAs

Table 4 shows the quality of the PRISMA 2020 report. Among the 27 included items, the reporting eligibility of 11 items was 100%, while the reporting eligibility of 11 items was at least 66.67%, indicating that most of the reports were relatively complete. However, there were five substantive deficiencies in the report: Abstract (Item 2), evidence quality evaluation (Item 15), evidence quality evaluation results (Item 22), registration and agreement (Item 24), and data disclosure (Item 27). Only one SR/MA (16.7%) reported retrieval strategies and information availability. Only two SRs/MAs (33.3%) reported supporting data. The remaining entries were only partially completed.

**Table 4:** Report quality evaluation included in SRs/MAs by PRISMA 2020

**Topic**		**Item number**	**Yang AL 2018 **[29]	**Wu JH 2019 **[8]	**Liu SY 2018 **[6]	**Lv YH 2015 **[30]	**Li Y 2016 **[31]	**Zhang HB 2019 **[7]	**Number of y (%)**
Title	Title	Item 1	y	y	y	y	y	y	6(100%)
Abstract	Abstract	Item 2	n	py	py	py	py	py	0(0)
Introduction	Rationale basis	Item 3	y	y	y	y	y	y	6(100%)
	Objectives	Item 4	y	y	y	y	y	y	6(100%)
Methods	Eligibility criteria	Item 5	y	py	y	y	y	py	4(66.67%)
	Information sources	Item 6	py	py	y	py	y	y	3(50%)
	Search strategy	Item 7	n	n	y	n	n	n	1(16.7%)
	Screening process	Item 8	y	y	y	y	y	y	6(100%)
	Data extraction	Item 9	n	n	y	y	y	y	4(66.67%)
	Data items	Item 10a	y	y	y	py	py	py	3(50%)
		Item 10b	y	y	y	y	y	y	6(100%)
	Study risk of bias assessment	Item 11	y	y	y	y	y	y	6(100%)
	Effffect measures	Item 12	y	y	y	y	y	y	6(100%)
	Synthesis methods	Item 13a	y	y	y	y	y	y	6(100%)
		Item 13b	y	y	y	y	y	y	6(100%)
		Item 13c	y	y	y	y	y	y	6(100%)
		Item 13d	y	y	y	y	y	y	6(100%)
		Item 13e	n	n	y	n	y	y	3(50%)
		Item 13f	n	y	y	n	n	y	3(50%)
	Reporting bias assessment	Item 14	n	y	y	y	n	y	4(66.67%)
	Quality of outcome evidence	Item 15	n	n	n	n	n	n	0(0)
Results	Study selection	Item 16a	n	y	y	y	y	y	5(83.3%)
		Item 16b	n	y	y	y	y	y	5(83.3%)
	Study characteristics	Item 17	y	y	y	y	y	y	6(100%)
	Risk of bias in studies	Item 18	y	y	y	y	y	y	6(100%)
	Results of individual studies	Item 19	y	y	y	y	y	y	6(100%)
	Results of syntheses	Item 20a	py	y	y	y	y	y	5(83.3%)
		Item 20b	y	y	y	y	y	y	6(100%)
		Item 20c	n	y	y	n	y	y	4(66.67%)
		Item 20d	n	y	y	n	y	y	4(66.67%)
	Reporting bias	Item 21	n	y	y	y	n	y	4(66.67%)
	Certainty of evidence	Item 22	n	n	n	n	n	n	0(0)
Discussion	Discussion	Item 23a	y	y	y	y	y	y	6(100%)
		Item 23b	y	n	y	y	y	y	5(83.3%)
		Item 23c	y	n	y	y	y	y	5(83.3%)
		Item 23d	y	y	y	y	y	y	6(100%)
Other Information	Registration and protocol	Item 24a	n	n	n	n	n	n	0(0)
		Item 24b	n	n	n	n	n	n	0(0)
		Item 24c	n	n	n	n	n	n	0(0)
	Supporting	Item 25	n	n	y	y	n	n	2(33.3%)
	Statement of Conflict of Interest	Item 26	n	n	y	y	y	y	4(66.67%)
	Information Availability	Item 27	n	n	n	n	n	y	1(16.7%)

### Risk of bias of included SRs/MAs

Table 5 shows the bias risk assessment of the overview. In Phase 1 and Domain 1 in Phase 2, the six SRs/MAs were rated as low risk of bias, which evaluated eligibility criteria and identification and selection of studies. In Domain-2, two studies [8, 29] had a high risk of bias because the retrieval database was incomplete or the screening process was not provided. In Domain-3, one study [29] was rated as high bias risk because it did not mention the data extraction process, and one study [8] did not involve double reviewers in the data extraction process. In Domain-4, two studies [29, 30] did not deal with heterogeneity or conduct sensitivity analysis, which had a high risk of bias. In Phase-3, the three studies [29–31] did not analyze or deal with the bias in SRs/MAs, which had a high risk of bias.

**Table 5:** Risk of bias included in SRs/MAs by ROBIS

**Phase**	**Production process**	**Yang AL 2018 **[29]	**Wu JH 2019 **[8]	**Liu SY 2018 **[6]	**Lv YH 2015 **[30]	**Li Y 2016 **[31]	**Zhang HB 2019 **[7]
Phase1	Study eligibility criteria	Low Risk	Low Risk	Low Risk	Low Risk	Low Risk	Low Risk
Phase 2	①Identification and selection of studies	Low Risk	Low Risk	Low Risk	Low Risk	Low Risk	Low Risk
	②Data collection and study appraisal	HIgh Risk	HIgh Risk	Low Risk	unclear risk	Low Risk	Low Risk
	③Data extraction and quality evaluation	HIgh Risk	HIgh Risk	Low Risk	Low Risk	Low Risk	Low Risk
	④Data synthesis and result presentation	HIgh Risk	Low Risk	Low Risk	HIgh Risk	Low Risk	Low Risk
Phase 3	Risk of bias in the review	HIgh Risk	Low Risk	Low Risk	HIgh Risk	HIgh Risk	Low Risk

### Quality of evidence in the included SRs/MAs

Table 6 shows the 42 results of GRADE assessments for six SRs/MAs; 1 (1/42, 2.38%), 17 (17/42, 40.48%), and 24 (24/42, 57.14%) were rated moderate quality, low quality, and very low quality, respectively. No high-quality evidence results were found. The main reason for the low quality of evidence was the design of RCTs (42/42, 100.00%), which was the most important factor leading to the lower quality of evidence, followed by inconsistency (19/42, 45.24%), publication bias (41/42, 97.62%), and inaccuracy (3/42, 7.14%). Efficacy was the most commonly used outcome indicator, with six SRs/MAs reporting validity, including one moderate level of evidence, four lower level of evidence, and one very lower level of evidence. The KPS score is a commonly used indicator for evaluating the quality of life of patients with BC, and the five included SRs/MAs reported KPS, including results from four low-level evidence and one moderate level of evidence. Safety was an important outcome of the SFI evaluation, and included the results of nine low-level evidence and one very low-level evidence studies.

**Table 6:** Quality of evidence in the included SRs/MAs based on GRADE

**References**	**Outcomes(participants)**	**Certainty assessment**						**Interventions**		**Effect**		**Certainty**
								**Test group**	**Control group**	**Relative(95% CI)**	**Absolute(95% CI)**	
		**Study design (number)**	**Risk of bias**	**Inconsistency**	**Indirectness**	**Imprecision**	**Other considerations**					
Yang AL 2018 [29]	RR	RCT (5)	serious^a^	not serious	not serious	not serious	serious^d^	151/211 ( 71.6%)	106/202 ( 52.5%)	RR 1.37 (1.17 to 1.60)	194 more per 1000 (from 89 more to 315 more)	⨁⨁◯◯Low
	CD3+	RCT (5)	serious^a^	serious^b^	not serious	not serious	serious^d^	209	197		SMD 0.63 higher (0.15 higher to 1.11 higher)	⨁◯◯◯Very low
	CD4+	RCT (7)	serious^a^	serious^b^	not serious	not serious	serious^d^	281	261		SMD 1.36 higher (0.59 higher to 2.14 higher)	⨁◯◯◯Very low
	CD8+	RCT (7)	serious^a^	serious^b^	not serious	not serious	serious^d^	281	261		SMD 0.07 lower (0.4 lower to 0.25 higher)	⨁◯◯◯Very low
	CD4+/CD8+	RCT (7)	serious^a^	serious^b^	not serious	not serious	serious^d^	281	261		SMD 0.56 higher (0.26 higher to 0.87 higher)	⨁◯◯◯Very low
	NK cell	RCT (3)	serious^a^	serious^b^	not serious	not serious	serious^d^	143	131		SMD 1 higher (0.39 higher to 1.61 higher)	⨁◯◯◯Very low
Wu JH 2019 [8]	RR	RCT (11)	serious^a^	not serious	not serious	not serious	not serious	325/510 ( 63.7%)	223/496 ( 45.0%)	RR 1.41 (1.26 to 1.58)	184 more per 1000 (from 117 more to 261 more)	⨁⨁⨁◯Moderate
	KPS score	RCT (10)	serious^a^	not serious	not serious	not serious	serious^d^	266/592 ( 44.9%)	129/573 ( 22.5%)	RR 1.95 (1.65 to 2.31)	214 more per 1000 (from 146 more to 295 more)	⨁⨁◯◯Low
	CD3+	RCT (7)	serious^a^	serious^b^	not serious	not serious	serious^d^	311	297		SMD 1.5 higher (0.52 higher to 2.48 higher)	⨁◯◯◯Very low
	CD4+	RCT (9)	serious^a^	serious^b^	not serious	not serious	serious^d^	365	346		SMD 2.42 higher (1.42 higher to 3.41 higher)	⨁◯◯◯Very low
	CD8+	RCT (9)	serious^a^	serious^b^	not serious	not serious	serious^d^	373	351		SMD 0.32 lower (1.11 lower to 0.46 higher)	⨁◯◯◯Very low
	CD4+/CD8+	RCT (9)	serious^a^	serious^b^	not serious	not serious	serious^d^	373	351		SMD 0.32 lower (1.11 lower to 0.46 higher)	⨁◯◯◯Very low
	Th-cell	RCT (4)	serious^a^	not serious	not serious	serious^c^	serious^d^	231	226		SMD 3.4 higher (1.52 higher to 5.29 higher)	⨁◯◯◯Very low
	Thrombocytopenia	RCT (7)	serious^a^	not serious	not serious	not serious	serious^d^	357	342		SMD 0.29 higher (0.08 higher to 0.49 higher)	⨁⨁◯◯Low
	Leukopenia	RCT (6)	serious^a^	not serious	not serious	not serious	serious^d^	120/393 ( 30.5%)	192/388 ( 49.5%)	RR 0.62 (0.52 to 0.74)	188 fewer per 1000 (from 238 fewer to 129 fewer)	⨁⨁◯◯Low
Liu SY 2018 [6]	RR	RCT (13)	serious^a^	not serious	not serious	not serious	serious^e^	390/577 ( 67.6%)	299/573 ( 52.2%)	RR 1.30 (1.18 to 1.43)	157 more per 1000 (from 94 more to 224 more)	⨁⨁◯◯Low
	KPS score	RCT (15)	serious^a^	not serious	not serious	not serious	serious^d^	278/534 ( 52.1%)	120/505 ( 23.8%)	RR 2.18 (1.84 to 2.57)	280 more per 1000 (from 200 more to 373 more)	⨁⨁◯◯Low
	Leukopenia	RCT (12)	serious^a^	not serious	not serious	not serious	serious^d^	85/449 ( 18.9%)	164/440 ( 37.3%)	RR 0.51 (0.41 to 0.63)	183 fewer per 1000 (from 220 fewer to 138 fewer)	⨁⨁◯◯Low
	Gastrointestinal reaction	RCT (8)	serious^a^	not serious	not serious	not serious	serious^d^	60/304 ( 19.7%)	117/304 ( 38.5%)	RR 0.51 (0.40 to 0.66)	189 fewer per 1000 (from 231 fewer to 131 fewer)	⨁⨁◯◯Low
	Hepatorenal dysfunction	RCT (7)	serious^a^	not serious	not serious	not serious	seriousd	24/298 ( 8.10%)	64/298 ( 21.5%)	RR 0.38 (0.25 to 0.59)	133 fewer per 1000 (from 161 fewer to 88 fewer)	⨁⨁◯◯Low
Lv YH 2015 [30]	RR	RCT (8)	serious^a^	not serious	not serious	not serious	serious^e^	193/303 ( 63.7%)	140/293 ( 47.8%)	RR 1.33 (1.15 to 1.53)	158 more per 1000 (from 72 more to 253 more)	⨁⨁◯◯Low
	KPS score	RCT (8)	serious^a^	not serious	not serious	not serious	serious^d^	236/285 ( 82.8%)	145/260 ( 55.8%)	RR 1.44 (1.20 to 1.73)	245 more per 1000 (from 112 more to 407 more)	⨁⨁◯◯Low
	CD3+	RCT (5)	serious^a^	serious^b^	not serious	not serious	serious^d^	228	214		SMD 1.33 higher (0.18 higher to 2.49 higher)	⨁◯◯◯Very low
	CD4+	RCT (6)	serious^a^	serious^b^	not serious	not serious	serious^d^	260	238		SMD 1.72 higher (0.61 higher to 2.84 higher)	⨁◯◯◯Very low
	CD8+	RCT (6)	serious^a^	serious^b^	not serious	not serious	serious^d^	260	238		SMD 0.19 higher (0.51 lower to 0.89 higher)	⨁◯◯◯Very low
	CD4+/CD8+	RCT (6)	serious^a^	serious^b^	not serious	not serious	serious^d^	260	238		SMD 0.62 higher (0.25 higher to 0.98 higher)	⨁◯◯◯Very low
	NK cell	RCT (4)	serious^a^	serious^b^	not serious	not serious	serious^d^	198	184		SMD 1.09 higher (0.65 higher to 1.53 higher)	⨁◯◯◯Very low
	Leukopenia	RCT (8)	serious^a^	not serious	not serious	not serious	serious^e^	50/250 ( 20.0%)	108/248 ( 43.5%)	RR 0.45 (0.34 to 0.60)	240 fewer per 1000 (from 287 fewer to 174 fewer)	⨁⨁◯◯Low
	Thrombocytopenia	RCT (5)	serious^a^	not serious	not serious	not serious	serious^e^	20/169 ( 11.8%)	42/168 ( 25.0%)	RR 0.48 (0.30 to 0.76)	130 fewer per 1000 (from 175 fewer to 60 fewer)	⨁⨁◯◯Low
	Gastrointestinal reaction	RCT (7)	serious^a^	not serious	not serious	not serious	serious^e^	66/238 ( 27.7%)	142/232 ( 61.2%)	RR 0.45 (0.36 to 0.56)	337 fewer per 1000 (from 392 fewer to 269 fewer)	⨁⨁◯◯Low
	Electrocardiogram	RCT (5)	serious^a^	not serious	not serious	not serious	serious^e^	16/171 ( 9.4%)	40/160 ( 25.0%)	RR 0.36 (0.22 to 0.61)	160 fewer per 1000 (from 195 fewer to 98 fewer)	⨁⨁◯◯Low
Li Y 2016 [31]	RR	RCT (5)	serious^a^	serious^b^	not serious	not serious	serious^d^	175/265 ( 66.0%)	138/261 ( 52.9%)	RR 1.29 (1.04 to 1.61)	153 more per 1000 (from 21 more to 323 more)	⨁◯◯◯Very low
	KPS score	RCT (8)	serious^a^	not serious	not serious	not serious	serious^d^	253/321 ( 78.8%)	143/289 ( 49.5%)	RR 1.51 (1.34 to 1.71)	252 more per 1000 (from 168 more to 351 more)	⨁⨁◯◯Low
	Weight gain	RCT (2)	serious^a^	serious^b^	not serious	serious^c^	serious^d^	93/113 ( 82.3%)	32/105 ( 30.5%)	RR 2.70 (1.62 to 4.52)	518 more per 1000 (from 189 more to 1000 more)	⨁◯◯◯Very low
	Reduction of fatigue	RCT (2)	serious^a^	serious^b^	not serious	serious^c^	serious^d^	23/66 ( 34.8%)	52/66 ( 78.8%)	RR 0.44 (0.24 to 0.79)	441 fewer per 1000 (from 599 fewer to 165 fewer)	⨁◯◯◯Very low
Zhang HB 2019 [7]	RR	RCT (24)	serious^a^	not serious	not serious	not serious	serious^e^	739/1110 ( 66.6%)	541/1096 ( 49.4%)	RR 1.35 (1.26 to 1.45)	173 more per 1000 (from 128 more to 222 more)	⨁⨁◯◯Low
	KPS score	RCT (19)	serious^a^	serious^b^	not serious	not serious	serious^e^	668/949 ( 70.4%)	380/901 ( 42.2%)	RR 1.56 (1.35 to 1.81)	236 more per 1000 (from 148 more to 342 more)	⨁◯◯◯Very low
	CD3+	RCT (11)	serious^a^	serious^b^	not serious	not serious	serious^d^	484	474		SMD 0.97 higher (0.24 higher to 1.71 higher)	⨁◯◯◯Very low
	CD4+	RCT (14)	serious^a^	serious^b^	not serious	not serious	serious^d^	587	569		SMD 1.66 higher (0.95 higher to 2.38 higher)	⨁◯◯◯Very low
	CD8+	RCT (14)	serious^a^	serious^b^	not serious	not serious	serious^d^	587	569		SMD 0.04 higher (0.59 lower to 0.67 higher)	⨁◯◯◯Very low
	CD4+/CD8+	RCT (14)	serious^a^	serious^b^	not serious	not serious	serious^d^	587	569		SMD 0.8 higher (0.38 higher to 1.22 higher)	⨁◯◯◯Very low
	NK cell	RCT (10)	serious^a^	serious^b^	not serious	not serious	serious^d^	485	458		SMD 0.94 higher (0.63 higher to 1.24 higher)	⨁◯◯◯Very low

^a^There were large deviations in randomization, assignment concealment or blinding

^b^The heterogeneity test P was very small, and the I2 was larger

^c^The sample size is small, or (and) the CI, is wide

^d^A funnel diagram analysis was not conducted

^e^A funnel plot showed publication bias

### Results of data synthesis and quantitative analysis

#### Clinical response rate

**Fig. 4:**
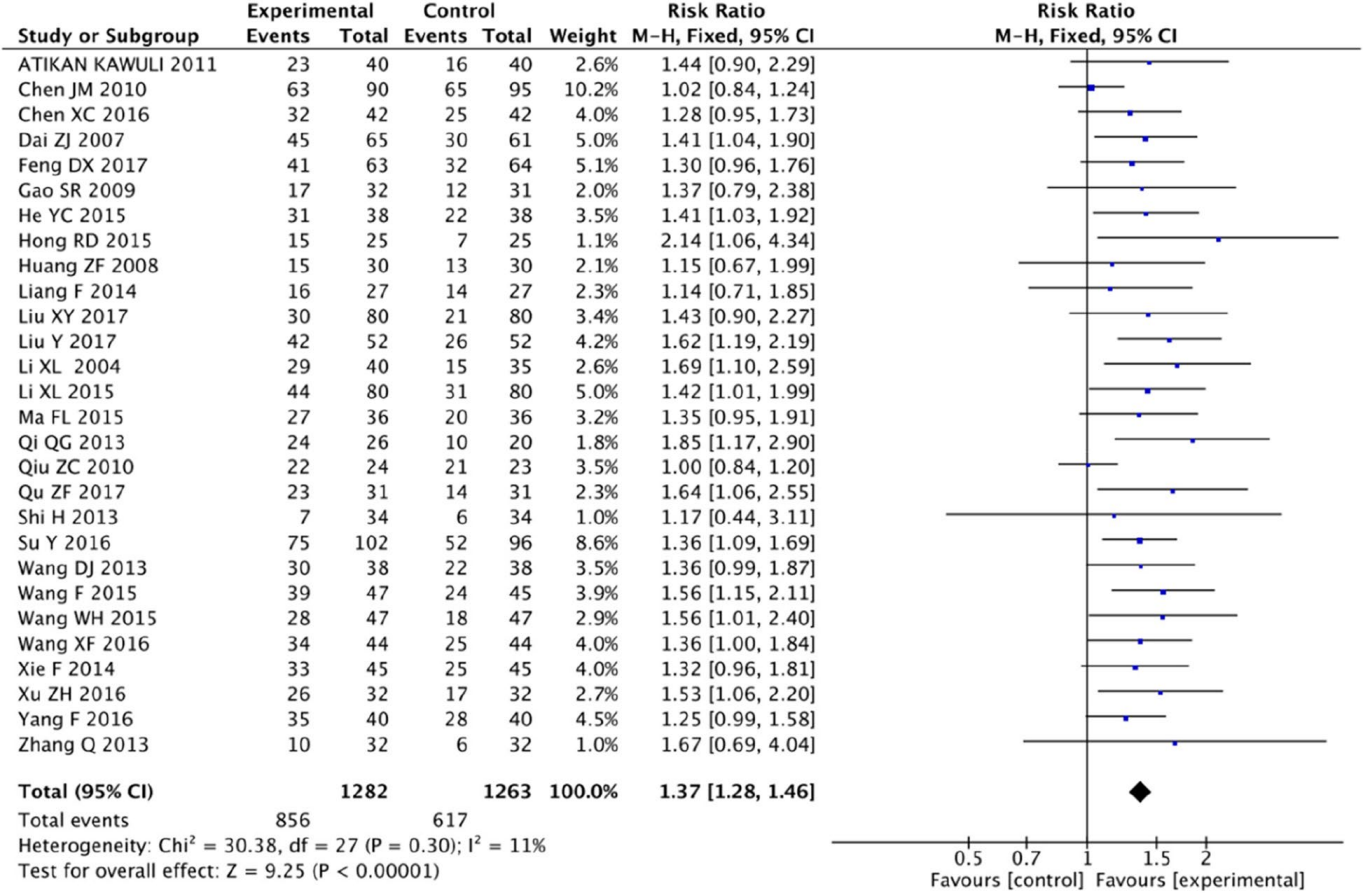
Forest plot of clinical response rate of SFI for BC

The overview conducted a meta-analysis of the RCTs included in six SRs/MAs. After screening and removing duplicates, 28 RCTs (2545 participants) reported a clinical response rate. The heterogeneity between the studies was small (*P=*0.30, I^2=^11%), and a fixed effect model was used. The results showed that the clinical response rate of the adjuvant CT group of SFI was better than that of the CT group (RR=1.37, 95% CI 1.28, 1.46; *P<*0.00001) (Fig. 4).

Publication bias analysis was performed using a funnel plot, and the results showed asymmetry, which indicated the low quality of the included studies and the small sample size. (Supplementary material 2, Figure S1).

### KPS score

A meta-analysis of KPS scores was performed on 24 RCTs (1890 participants) included in the six SRs/MAs. There was a large heterogeneity between the studies (*P*<0.0001, I^2^=70%). A random effect model was used. The KPS score of the adjuvant CT group of SFI was better than that of the CT group (RR=1.58, 95% CI 1.39, 1.80; *P*<0.00,001). (Supplementary material 2, Figure S2).

The KPS score had a large heterogeneity (I^2^=70%). After the comparative analysis of the literature, we removed the research of Song 2004 [33], Su 2016 [34], and Xu 2010 [35], and the heterogeneity of the KPS score decreased significantly (I^2^=27%). Therefore, we considered that the heterogeneity of the KPS score was mainly related to the low quality of the included studies. (Supplementary material 2, Figure S3).

### Immune function

A meta-analysis of CD3+ assessment was performed on 13 RCTs (1124 participants) in the six SRs/MAs included and showed that there was a large heterogeneity between the studies (*P<*0.0001, I^2=^96%), and a random effect model was used. The results showed that CD3 + of the SFI adjuvant CT group was better than that of the CT group (SMD=1.51, 95% CI 0.91, 2.10; *P*<0.00001).

Seventeen RCTs (1382 participants) reported the level of CD4+, which was highly heterogeneous among the studies (*P<*0.0001, I^2=^97%). A random effect model was used. The results showed that CD4 + from the adjuvant CT group of SFI was better than that of the CT group (SMD=1.87, 95% CI 1.18, 2.56; *P*<0.00001).

Seventeen RCTs (1382 participants) reported the level of CD8+, which was highly heterogeneous among the studies (*P<*0.0001, I^2=^96%). The results showed that CD8+ of the adjuvant CT group of SFI was similar to that of the CT group, and the difference was not statistically significant (SMD=-0.21, 95% CI -0.82, 0.41; *P=*0.51).

Seventeen RCTs (1382 participants) reported the level of CD4+/CD8+, which was highly heterogeneous among the studies (*P<*0.0001, I^2=^91%). The results showed that the CD4+/CD8+ of the SFI adjuvant CT group was better than that of the CT group (SMD=0.86, 95% CI 0.48, 1.23; *P*<0.00001).

**Fig. 5:**
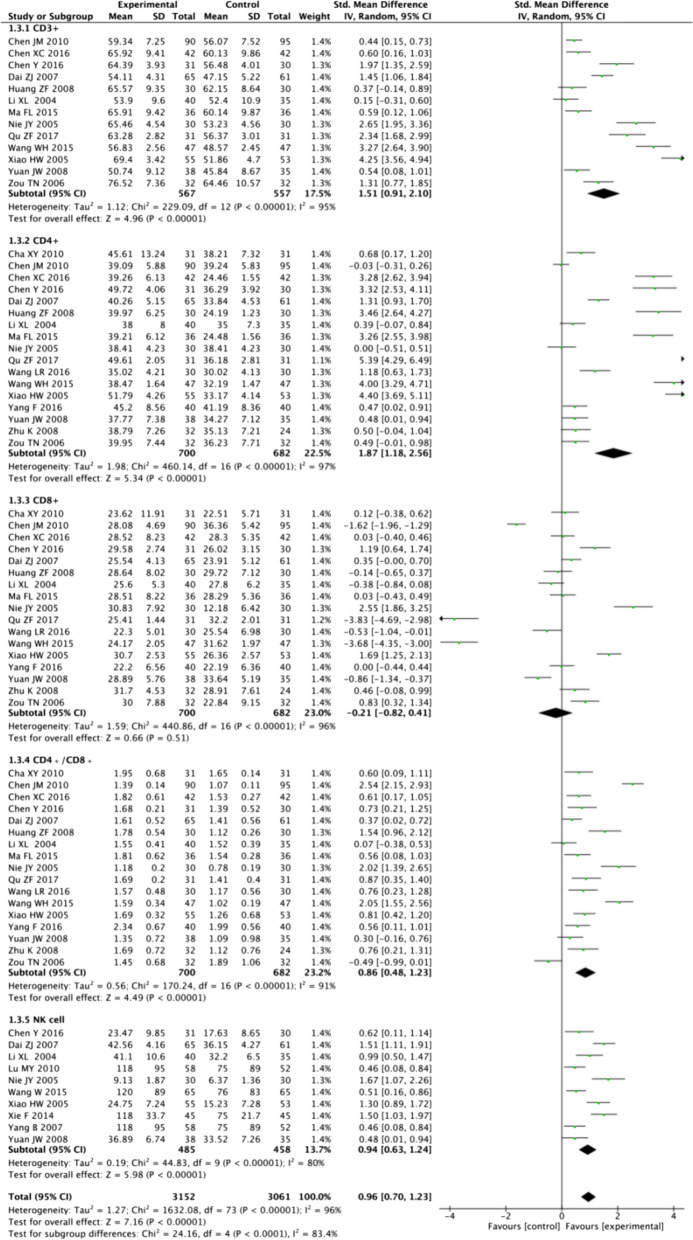
Forest plot of immune function

Ten RCTs (943 participants) reported the level of NK cells, which was highly heterogeneous among the studies (*P<*0.0001, I^2=^80%). The results showed that the NK cells of the SFI adjuvant CT group were better than those of the CT group (SMD=0.94, 95% CI 0.63, 1.24; *P*<0.00001) (Fig. 5).

### Adverse reactions

A meta-analysis of leukopenia was conducted in seventeen RCTs (1480 participants) in the six included SRs/MAs, which that showed that there was no heterogeneity among the studies (*P=*0.80, I^2=^0%), and a fixed effect model was used. The results indicated that the incidence of leukopenia in the adjuvant CT group of SFI was lower than in the CT group (RR=0.53, 95% CI 0.46, 0.62; *P*<0.00001).

**Fig. 6:**
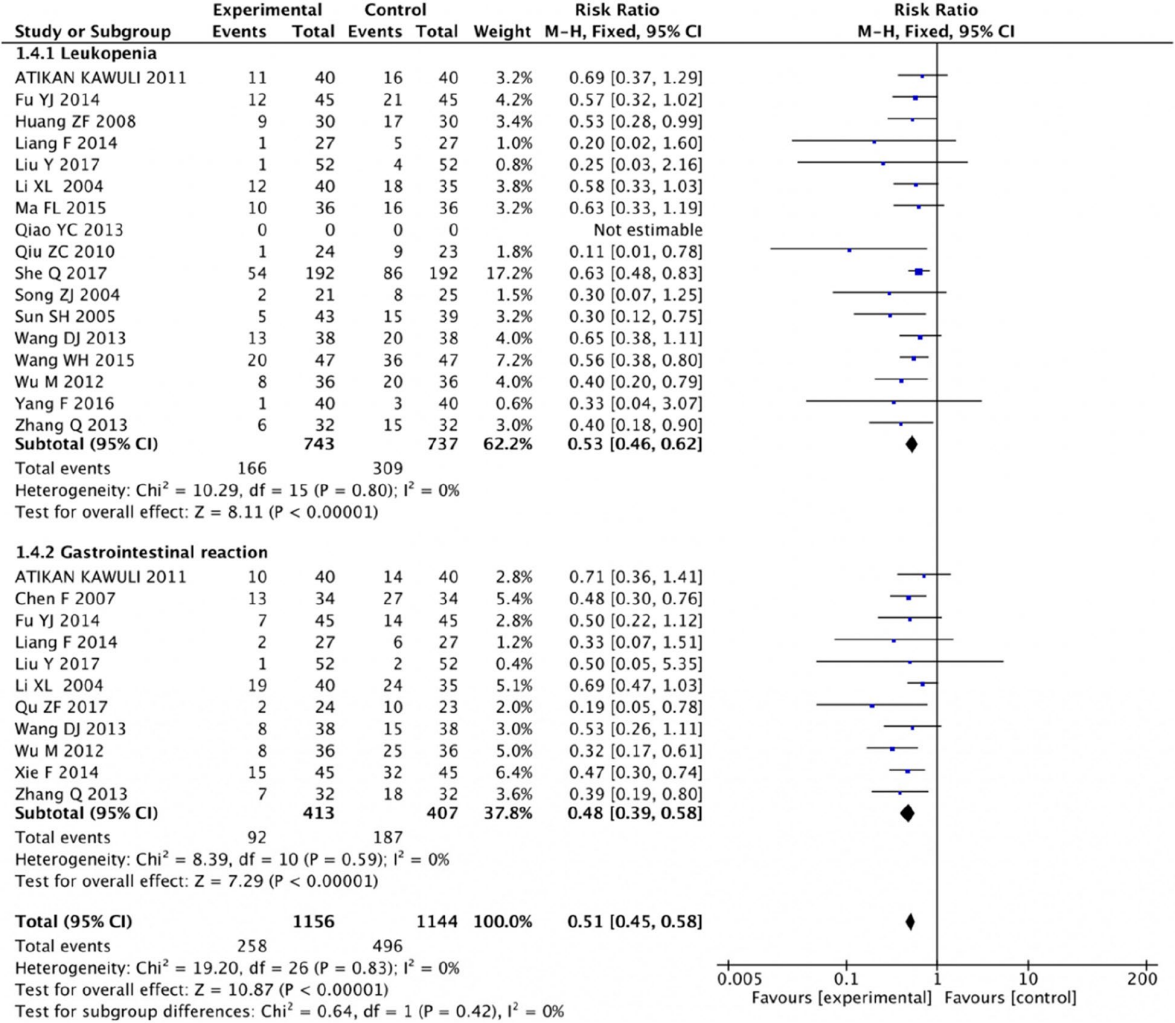
Forest plot of adverse reactions

Eleven RCTs (820 participants) reported the gastrointestinal reactions, which did not show heterogeneity between studies (*P=*0.59, I^2=^0%). The incidence of gastrointestinal reaction of the adjuvant CT group of SFI was lower than that of the CT group (RR=0.48, 95% CI 0.39, 0.58; *P*<0.00001) (Fig. 6).

## Discussion

In China, patients with BC accept adjuvant treatment with TCM, such as SFI, due to the unsatisfactory efficacy achieved following CT, due to decreasing quality of life or side effects [5]. This has aroused the interest of researchers, and many related RCTs have been conducted. Previous studies have shown that adjuvant SFI CT therapy seems to have achieved good efficacy, while also achieving good safety [30]. Related SRs/MAs have also been published more frequently, but there is still controversy about the clinical efficacy and safety of SFI in the treatment of breast cancer. For example, conclusions about improving immune function and protecting myelosuppression are not consistent [8, 30]. Therefore, we conducted this review by searching for SRs/MAs of all corresponding RCTs evaluating the treatment of SFI for BC, using AMSTAR-2, PRISMA 2020, ROBIS, and GRADE tools to assess the level of quality evidence and clarify how SFI benefits BC.

### Summary of findings

In this overview, the evidence for the efficacy and safety of SFI in the treatment of BC was derived from six SRs/MAs. The results of the quantitative meta-analysis found that adjuvant CT of SFI in the treatment of patients with BC was beneficial, specifically in improving the clinical response rate, quality of life, regulating immune function of the body, and with fewer leukopenia and gastrointestinal reactions. However, the overall methodological quality and evidence quality of the original RCTs included in these SRs/MAs were generally poor, lacking large-scale multicenter, placebo-controlled studies, resulting in almost all included SRs/MAs being unable to draw firm and reliable conclusions on the efficacy and safety of adjuvant CT treatment with SFI for BC. As shown by the results of the evaluation of AMSTAR-2, PRISMA 2020, ROBIS, and GRADE, the methods and quality of the evidence for most SRs/MAs are not satisfactory. Therefore, there is an urgent need to further improve the design of RCTs and SRs/MAs research protocols to determine the true efficacy and safety of SFI in the treatment of BC.

Evidence rated as high quality or moderate quality is considered relatively reliable [36]. The methodological quality of six SRs/MAs studies was evaluated by AMSTAR-2, among which three studies were of very low quality and three studies were of low quality, no high quality, or moderate quality. Although six SRs/MAs followed the PICO principle and used double reviewers to detect and assess the risk of bias in each included study and applied appropriate statistical methods, methodological deficiencies include the failure to publish predesigned study protocols, the failure to provide a detailed list of reasons for excluding studies, and the failure to report funding sources for inclusion in RCTs. Similar to the results of AMSTAR-2, the PRISMA 2020 evaluation showed that the included SRs/MAs also had these deficiencies. Furthermore, six SRs/MAs had different degrees of information deficiencies in the literature retrieval, data extraction, and description, discussion of bias risk, interest statements, and information disclosure. In the ROBIS, further analysis found insufficient explanations for the risk of bias, the risk of identifying and selecting bias in research, and insufficient evaluation of publication bias were the main factors leading to a high risk of bias. In summary, the unsatisfactory results of AMSTAR-2, PRISMA 2020, and ROBIS were mainly limited to the registration of the study protocols, the explanation of the basis for the inclusion of only RCTs, the description of the funding sources for each included study, the incomplete search strategy and screening process, addressing of heterogeneity and sensitivity, and reporting of potential conflicts of interest.

The overview evaluated the quality of the evidence of the outcome indicators using GRADE. Our results indicated that adjuvant SFI CT is beneficial for outcome indicators such as response rate, KPS score, CD3+, CD4+, CD4+/CD8+, NK cells, leukopenia, and gastrointestinal reactions in patients with BC. However, it is worth noting that the quality level of this evidence varies from ‘moderate’ to ‘very low.’ The main reasons for the design of RCTs (42/42, 100.00%), inconsistency (19/42, 45.24%), publication bias (41/42, 97.62%), and inaccuracy (3/42, 7.14%). The main reasons for the poor quality of the study were the design of the RCTs, specifically the defects of randomization, assignment concealment, the implementation of blinded methodology, and the risk of selective reporting. Second, there was publication bias, or no bias test was conducted, and the source of bias analysis was not analyzed. Additionally, some studies were downgraded in terms of inconsistency due to the large heterogeneity of the included literature and the lack of analysis on the source of heterogeneity. Furthermore, due to the small sample size included in a few studies, the effect size of the combined outcome indicators showed a wide confidence interval, which contributed to a decrease in precision.

### Implications for future studies

In summary of the above deficiencies, the following implications should be applied to future SRs/MAs. First, whether it is SRs/MAs or RCTs, it is important to determine whether similar topics already exist or are ‘in progress’ before starting the study. Preregistration can maximize resource waste, reduce bias in the research process, and increase the authenticity, precision, and completeness of the study. The high overlap of CCA further reflected the unnecessary duplication of studies on the treatment of BC by adjuvant CT of SFI. Future SRs/MAs should only be conducted when the research objectives are significantly different. Preregistration of SRs/MAs may help avoid unnecessary duplications. Second, a comprehensive and detailed search strategy and indexing criteria should be formulated. The gray literature should not be ignored. Comprehensive, objective, and repeatable search and screening strategies are the basis for high-quality SRs/MAs. Third, the research should report on sources of funding and conflicts of interest. Fourth, all studies should explain the sources of heterogeneity and the risk of bias, and conduct subgroup and sensitivity analyses. Fifth, although RCT is the gold standard for clinical evidence, the specific reasons for choosing this type of study should also be explained. Furthermore, the principles of multi-person independence, repetition, strict screening, and data extraction must also be strictly implemented. Based on the above, the quality of SRs/MAs can be improved.

The GRADE analysis of the included studies indicated that for most outcome measures, the quality of evidence was low or very low. It is mainly the design of RCTs that leads to degradation of study quality. Future studies should focus on the design of high-quality RCTs with large sample sizes and placebo-controlled controls. Another factor that leads to a low quality of evidence is inconsistency. Most studies had a high heterogeneity in outcome indicators, but no analysis was performed. In future SRs/MAs, subgroup analysis and sensitivity analysis should be conducted to identify the sources of heterogeneity. If heterogeneity cannot be reduced, descriptive analysis can be considered. Additionally, an insufficient sample size also leads to a decrease in accuracy. Finally, it should be mentioned that survival is an important outcome indicator for evaluating tumor patients. In RCTs research, there is a lack of research on SFI for the survival of patients with BC. Prolonging survival is the ultimate goal of adjuvant SFI treatment for BC, and future research should focus on evaluating survival-related indicators.

In general, existing evidence strongly shows that adjuvant SFI CT is beneficial for patients with BC. SFI, as a TCM injection, is widely used in the treatment of BC due to its good enhancement effect and reduction in toxicity. Its mechanism may be as follows: (1) improve immune cell function: SFI can regulate the level of T-cell subsets, enhance Th1 cells, and reduce Th2 cells to regulate their balance [37, 38]; (2) SFI can improve cisplatin drug sensitivity by regulating M2 tumor-associated macrophages (TAMs), reduce cisplatin-induced IL-10 and PGE2 release, and improve CT drug sensitivity [39]; (3) SFI can inhibit the proliferation of human BC MDA-MB-231 cells, block the cell cycle, and induce cell apoptosis by up-regulating the PUMA gene [40] and (4) SFI can protect bone marrow hemotopoietic function, enhance body sensitivity to granulocyte stimulating factor, protect white blood cells, and play a role in reducing toxicity and enhancing the antitumor effect [41]. Furthermore, network pharmacology studies have found that the SFI treatment can exert an antibreast cancer activity through nitrogen metabolism, adherent junctions, gap junctions, the HIF-1 signaling pathway, and other signaling pathways [42, 43].

### Limitations

This is the first overview study to examine the quality of evidence on the safety and efficacy of SFI for patients with BC using AMSTAR-2, PRISMA 2020, ROBIS, and GRADE. However, there were some limitations to our study. First, the pathological subtypes and clinical stages of patients with BC were not unified, and CT regimens were completely inconsistent. Second, all studies lacked long-term follow-up data to evaluate the long-term efficacy of SFI for patients with BC. Third, all evaluation tools used in the overview were adopted by skilled researchers, although the quality evaluation of the results was subjective. Fourth, all the RCTs in this study were conducted in China, which reduced the credibility of the results reporting.

## Conclusion

Although the overview confirmed the clinical value of SFI as an adjuvant CT in the treatment of BC through qualitative and quantitative analysis of the six SRs/MAs included in the analysis. However, in view of the poor methodological quality and evidence quality of most of the investigated SRs/MAs, the ability to definitively clarify the benefits of SFI in adjuvant treatment of BC is limited. Therefore, additional high-quality studies are needed to clarify the clinical significance of SFI in the treatment of BC.

### Supplementary Information

**Additional file 1: Supplementary material 1.**

**Additional file 2: Supplementary material 2.**

## Data Availability

All data and materials included in this study are included in this published article and supplementary material.
