# Supplementary material for: The role of Shenqi Fuzheng injection as adjuvant therapy for breast cancer: an overview of systematic reviews and meta-analyses
Source: BMC Complement Med Ther. 2024 Jan 11;24:33. doi: 10.1186/s12906-023-04274-4 (PMC10782532; doi:10.1186/s12906-023-04274-4)
Supplement: Supplementary file 1 — Additional file 1: Supplementary material 1. [file 12906_2023_4274_MOESM1_ESM.docx]

**Supplementary material 1**

Search strategy sample of Web of science

#1 ((TS=(breast cancer)) OR TS=(breast carcinoma)) OR TS=(breast neoplasm)

#2 ((TS=(shenqifuzheng)) OR TS=(shenqi fuzheng)) OR TS=(shenqi)

#3 ((TS=(systematic evaluation)) OR TS=(systematic review)) OR TS=(meta-analysis)

#4 #1 AND #2 AND #3

Search strategy sample of EMbase

#1 'breast cancer':ti,ab,kw OR 'breast carcinomc':ti,ab,kw OR 'breast cancer'

#2 'shenqifuzheng':ti,ab,kw OR 'shenqi fuzheng':ti,ab,kw OR 'SFI':ti,ab,kw

#3 'systematic evaluation':ti,ab,kw OR 'systematic review':ti,ab,kw OR 'meta-analysis

':ti,ab,kw

#4 #1 AND #2 AND #3

Search strategy sample of Cochrane Library

#1 (breast neoplasm) OR (breast carcinoma) OR (breast cancer)

#2 (shenqifuzheng) OR (shenqi fuzheng) OR (shenqi) OR (SFI)

#3 ((systematic review) OR (systematic evaluation)) OR (meta-analysis)

#4 #1 AND #2 AND #3

Search strategy sample of CNKI

SU=('shenqifuzheng'+'shenqifuzheng injection') AND SU=('breast cancer'+'breast carcinoma'+'breast neoplasm') AND SU=('systematic review'+'meta analysis'+'systematic evaluation')

SU: subject search.

Search strategy sample of Wanfang

subject:(“breast cancer” or “breast carcinoma” or “breast neoplasm”) and subject: (“shenqifuzheng” or “shenqifuzheng injection”) and subject: (“meta analysis” or “systematic evaluation”or “systematic review”)

Search strategy sample of VIP

U=(breast cancer+breast carcinoma+breast neoplasm) AND U= (shenqifuzheng+shenqifuzheng injection) AND U= (meta analysis+systematic evaluation+systematic review)
