# Supplementary material for: The role of Shenqi Fuzheng injection as adjuvant therapy for breast cancer: an overview of systematic reviews and meta-analyses
Source: BMC Complement Med Ther. 2024 Jan 11;24:33. doi: 10.1186/s12906-023-04274-4 (PMC10782532; doi:10.1186/s12906-023-04274-4)
Supplement: Supplementary file 2 — Additional file 2: Supplementary material 2. [file 12906_2023_4274_MOESM2_ESM.docx]

**Supplementary material 2**


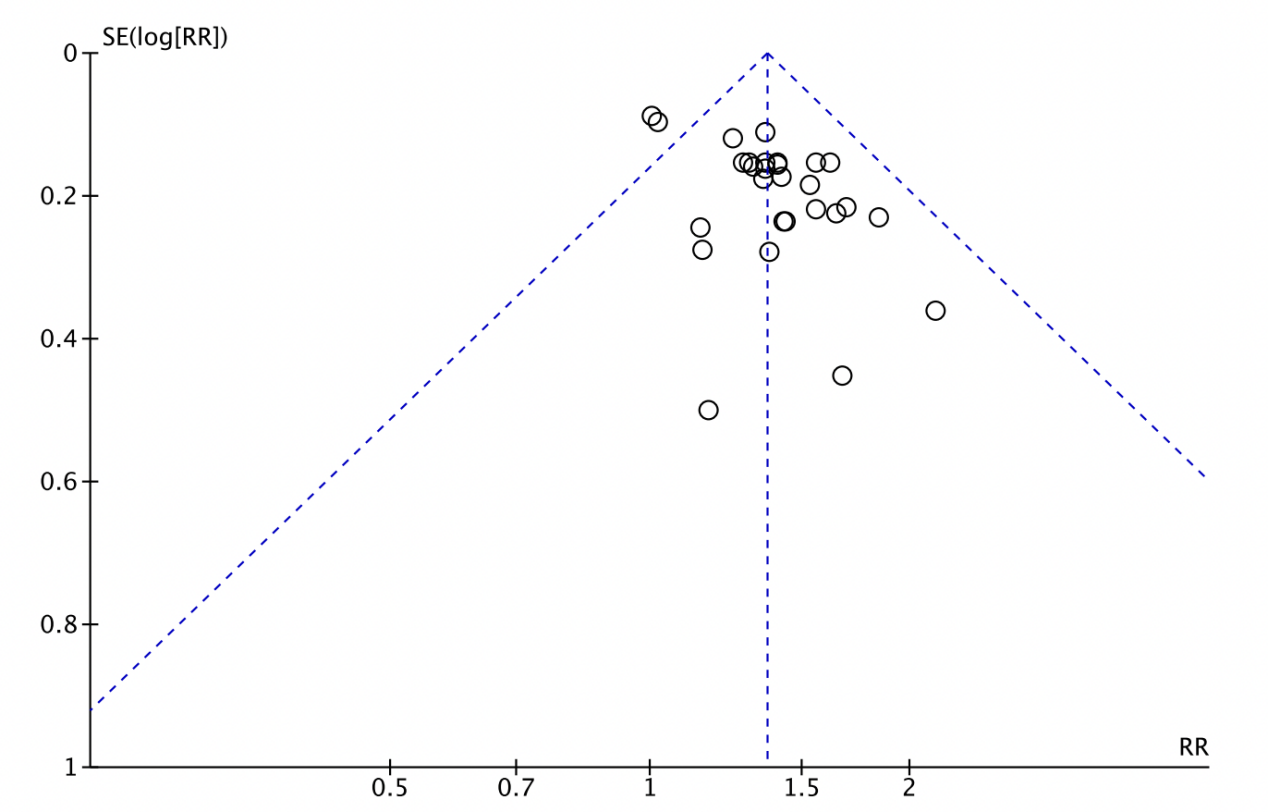


**Figure S1. Funnel plot of clinical response rate**

**
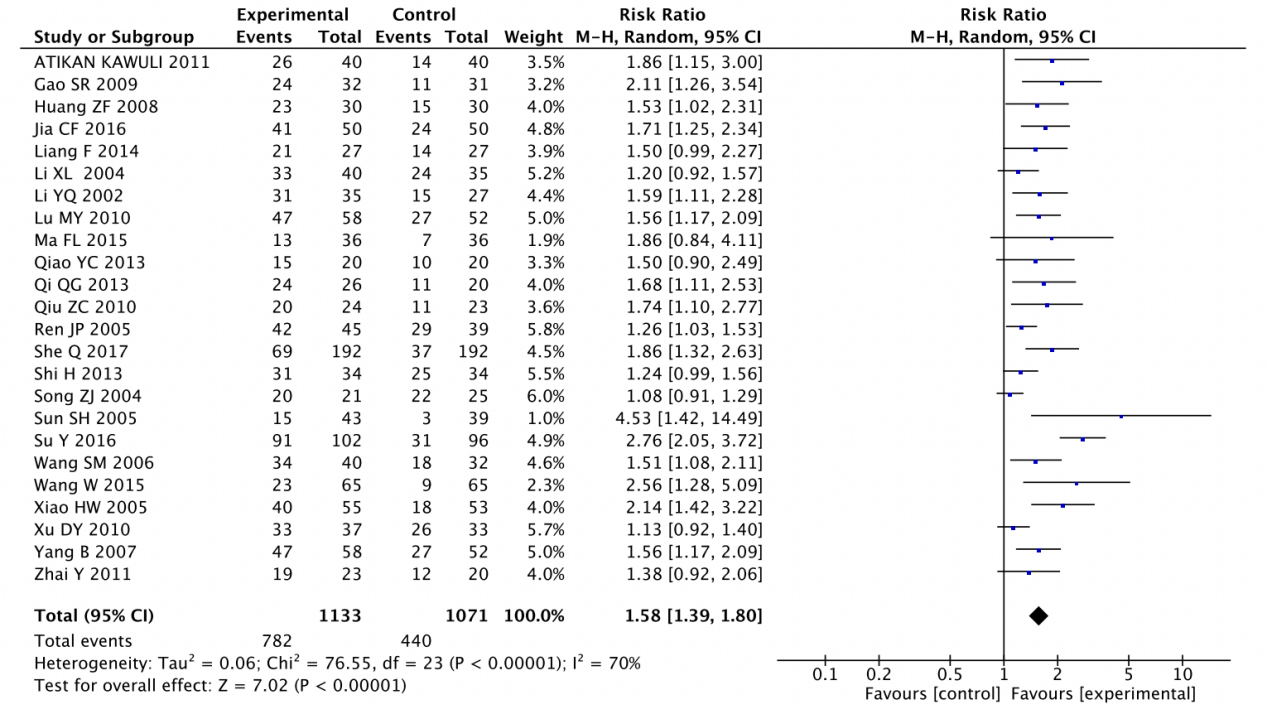
**

**Figure S2. Forest plot of KPS score**


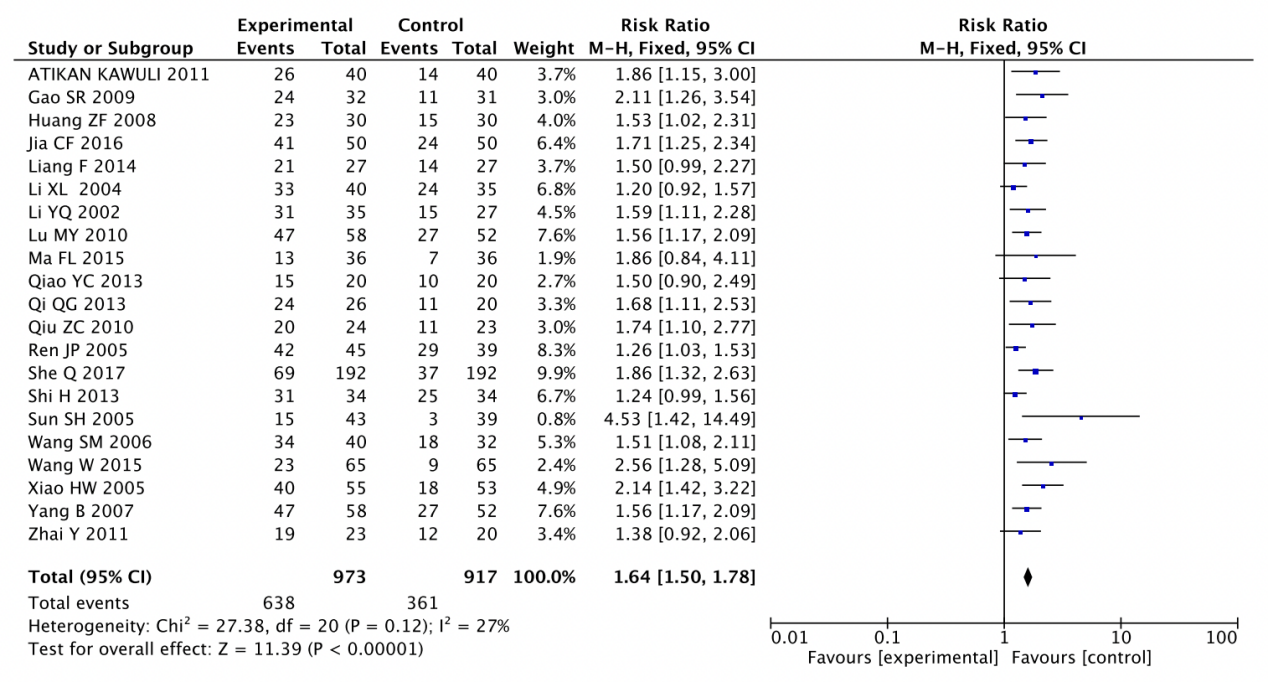


**Figure S3. Forest plot of sensitivity analysis of KPS score**
